# Editorial: Emerging talents in pharmacology of ion channels and channelopathies 2022

**DOI:** 10.3389/fphar.2023.1191328

**Published:** 2023-04-12

**Authors:** Daniel Yakubovich

**Affiliations:** Department of Neonatology, Sanz Medical Center, Laniado Hospital, Netanya, Israel

**Keywords:** ion channel, young scientist, early career, bibliometrics, impact

Various definitions of the term “early career scientist” exist in literature some include such high scientific ranks like associate professor. However still most early career are scientists which are in process of their training and just “find a taste” of near independent scientific enquiry. Lehman’s theory of creativity (Lehman, 2017) supported by Simonton’s creativity model (Simonton, 1984) predicts creativity and productivity pattern of a scientist through lifetime career to be a curvilinear function of age with peak near 40 years old. Furthermore, it was shown that collaboration with top scientists during the early years of scientific career can greatly influence future productivity of young researchers.

“Annus mirabilis” papers included the seminal papers about special relativity theory, theory of photoelectric effect, mass-energy equivalence and explanation of Brownian motion and were published by Albert Einstein in 1905 in Annalen der Physik when he was 26 years old undergraduate PhD student (receiving his official PhD degree in January 1906) (Kox, 1998). Alan Loyd Hodgkin being 38 years old fellow in Trinity College together with his 3 years younger trainee Andrew Fielding Huxley developed a theory of action potential (in years the famous Hodgkin-Huxley model) which was published in 5 articles in Journal of Physiology in 1952 (Schwiening, 2012). All three, above mentioned, scientists were awarded Noble Prize in years to come and made most substantial contribution to modern science.

Considering the mentioned above creating a platform for a young scientists to publish and to collaborate with higher rank and more advanced age researchers seems to be plausible idea for further stimulation of the development and the productivity of early career investigators. In current Research Topic in Frontiers of Pharmacology (Emerging Talents in Pharmacology of Ion Channels and Channelopathies) a platform dedicated to original works and also other types of scientific publications submitted by students as first authors was established. So far, published articles were downloaded 609 times and viewed 3,053 times. In conjunction to peer-review process undergraduate and early graduate students underwent as first or collaborating first authors, the level of exposure of their work to scientific community is expected to contribute to their identification as emerging leaders in the field and to allow the community to follow the aspiring careers of our emerging, talented researchers.
